# The Combined Effect of ZnO and CeO_2_ Nanoparticles on *Pisum sativum* L.: A Photosynthesis and Nutrients Uptake Study

**DOI:** 10.3390/cells10113105

**Published:** 2021-11-10

**Authors:** Elżbieta Skiba, Monika Pietrzak, Sława Glińska, Wojciech M. Wolf

**Affiliations:** 1 Institute of General and Ecological Chemistry, Lodz University of Technology, 90-924 Lodz, Poland; monika.pietrzak@dokt.p.lodz.pl (M.P.); wojciech.wolf@p.lodz.pl (W.M.W.); 2 Laboratory of Microscopic Imaging and Specialized Biological Techniques, Faculty of Biology and Environmental Protection, University of Lodz, 90-237 Lodz, Poland; slawa.glinska@biol.uni.lodz.pl

**Keywords:** combined effect, *Pisum sativum* L., nanoparticles, cerium oxide, zinc oxide, metal uptake, photosynthesis, hydroponic culture

## Abstract

Cerium oxide nanoparticles (CeO_2_ NPs) and zinc oxide nanoparticles (ZnO NPs) are emerging pollutants that are likely to occur in the contemporary environment. So far, their combined effects on terrestrial plants have not been thoroughly investigated. Obviously, this subject is a challenge for modern ecotoxicology. In this study, *Pisum sativum* L. plants were exposed to either CeO_2_ NPs or ZnO NPs alone, or mixtures of these nano-oxides (at two concentrations: 100 and 200 mg/L). The plants were cultivated in hydroponic system for twelve days. The combined effect of NPs was proved by 1D ANOVA augmented by Tukey’s post hoc test at *p* = 0.95. It affected all major plant growth and photosynthesis parameters. Additionally, HR-CS AAS and ICP-OES were used to determine concentrations of Cu, Mn, Fe, Mg, Ca, K, Zn, and Ce in roots and shoots. Treatment of the pea plants with the NPs, either alone or in combination affected the homeostasis of these metals in the plants. CeO_2_ NPs stimulated the photosynthesis rate, while ZnO NPs prompted stomatal and biochemical limitations. In the mixed ZnO and CeO_2_ treatments, the latter effects were decreased by CeO_2_ NPs. These results indicate that free radicals scavenging properties of CeO_2_ NPs mitigate the toxicity symptoms induced in the plants by ZnO NPs.

## 1. Introduction

Nowadays, synthetic nanomaterials (NMs) are finding applications in almost all material aspects of human life and are particularly vital for contemporary technology and medicine [1,2,3,4,5]. The most important are sustainable energy production and storage, electronic devices, catalysts, sensors and adhesives, high-quality petro- and agrochemicals [6,7,8,9,10,11,12,13,14]. Their widespread use raises fundamental questions related to the environment, pollution, and safety [15,16,17].

In the diverse world of nanomaterials metal oxides firmly occupy a unique position. Their global market in 2020 was worth almost USD 5.3 billion with several forecasts indicating its steady rise to USD 9.3 billion in 2027 [18]. Obviously, this growing stream of NMs cannot be completely isolated from soil, air, or water and finally will find its way to terrestrial living organisms. Therefore, complex interactions of nanoparticles (NPs) with the biota and their further environmental fate deserve additional studies.

In a number of comprehensive papers on interactions of nanoparticles with living organisms, the authors investigated the role of chemical composition, particle shape, size, and mechanisms of aggregation of NPs [19,20,21,22]. The beneficial or harmful effects of NPs were also examined. Owing to their unique properties, nanomaterials can be successfully used in fertilizers, pesticides, or as dedicated chemical carriers or sensors [23,24,25]. This increasing flux of diverse nanomaterials approaching soil and plant environments should not be left without comprehensive studies on their migrations, uptake, and toxicities. The emerging picture suggests dynamic processes which act in complicated matrices. Identification of interactions among various substances poses a real challenge.

Obviously, nanoparticles affect plant metabolism in a number of ways and finally introduce changes to plant physiology at cellular, organ, and individual plant levels [26,27,28,29]. In particular, photosynthesis—the essential, bioenergy-generating process [30]—may be either facilitated or hampered by nanoparticles [31,32,33,34,35,36]. As pointed out by Du et al. [37] and Tighe-Neira et al. [35], the latter may be conveniently examined by gas exchange parameters augmented with the contents of photosynthetic pigments. Unfortunately, results are rarely completely consistent and a wide range of plant responses to nanoparticles could be expected. Notably, metal oxide NPs usually alter the photosynthesis rate, photochemical fluorescence, and quantum yield in plants [37] with ZnO and CeO_2_ being among the most active. Mukherjee et al. [38] described the negative effect of ZnO NPs at soil concentrations 125–500 mg/L on chlorophyll activity in *Pisum sativum* L. It results from the substitution of the Mg atoms at chlorophyll centers by Zn which finally hampers the photosynthesis process. The opposite effect was observed by Reddy Pullagurala et al. [39] during cilantro (*Coriandrum sativum*) cultivation. Supplementation with 100 or 200 mg/L ZnO NPs induced photosynthetic pigments production and boosted photosynthesis.

The ambient character of nanoparticulate cerium oxide combined with its increasing abundance in the environment makes this substance a useful agent to study plant metabolism effects triggered by anthropogenic nanomaterials. In particular, Wu et al. [32] demonstrated that CeO_2_ NPs (nanoceria) are a reactive oxygen species (ROS) scavenger in leaf mesophyll cells and defend the chloroplast photosynthetic machinery from abiotic stresses. On the other hand, the negative effect of nanoceria on photosynthesis in soybean plants was investigated by Li et al. [40]. They pointed out several aspects which are at stake, namely: inhibited conversion efficiency of C5 to C3 in the Calvin–Benson cycle, destruction of thylakoid membranes, and reduced chlorophyll synthesis and activity. All these factors participate in the final plant destruction.

Regrettably, the most relevant works on the subject are aimed at particular types of nanoparticles. Investigations of combined, mutual effects induced by either mixture of NPs or their additives and stabilizers are quite scarce. In real ecosystems, nanoparticles rarely play a solo performance, which is especially important in modern efficient agriculture. Increasing pressure on massive food production for the rapidly growing population has prompted the development of new, smart, and efficient agrochemicals [41,42,43,44].

The fertilizing effect of CeO_2_ and ZnO NPs attracted the attention of several research groups. It is quite well documented that cerium oxide NPs can alleviate plant salinity stress, act as a catalyst in chlorophyll production, and in scavenging reactive oxygen species which stabilize the chloroplast structure and cell walls [45,46,47]. On the other hand, nanoparticulate zinc oxide may be used to counteract cadmium toxicity in wheat and elevate zinc concentrations in plants. Thus, it can be a useful agent for Zn biofortification in cereals plantations and help to overcome the well recognized hidden hunger in humans resulting from the deficiency of Zn in cereals [25]. In this respect, optimization of photosynthetic efficiency by nanoparticles is a highly promising approach for a smart increase of crop production [48].

Mixtures of nanoparticles that are present in the growth environment affect plant development in a different way than single species [49,50,51]. In particular, the combined, mutual interactions between ZnO and CeO_2_ are quite likely indeed. Both substances often coexist in all compartments of the environment and their simultaneous presence in the environment is more than likely. Our previous works on *Pisum sativum* L. were related to metal migration strategies as induced by single stressors such as CeO_2_ or ZnO nanoparticles [52,53]. We have shown that zinc species alter Cu, Mn, and Fe uptake and their further migration in green pea. On the other hand, low concentrations of cerium oxide NPs increased the photosynthesis rate. Those investigations had a model character and did not account for combined effects as triggered by ZnO and CeO_2_ together in real plant matrices. This study enhances this approach significantly. Here, we examine the mixture of nanometric cerium and zinc oxides. The methodology has been based on hydroponic pot experiments [54]. Pea is frequently applied in system biology experiments and is treated as a non-model plant with a roughly complete genome structure [55,56]. It is cultivated worldwide [57] and additive interactions which affect nutrient uptake and enhance plant growth yield are of practical relevance.

## 2. Materials and Methods

### 2.1. Nanoparticles Characteristic

CeO_2_ NPs and ZnO NPs were purchased from the Byk (Byk-Chemie GmbH, Wesel, Germany) as commercially available products. The properties of nanoparticles, including average particle size, transmission electron microscopy (TEM) images, and zeta potential are given in Table S1 [52,54].

### 2.2. Plants Growth Conditions and Treatments

*Pisum sativum* L. plants were cultivated under hydroponic conditions in Hoagland’s nutrient solution. The composition of the growing medium and the detailed setup of the experiment were described previously [53,54]. The seeds, (“Iłówiecki” sugar pea, “PNOS” Co., Ltd., Ożarów Mazowiecki, Poland), sterilized in 70% ethanol were germinated in dark for 3 days. Next, the seedlings (at BBCH 09 phenological stage [58]) were transferred to perforated plastic plates and placed in containers with the nutrient solution. Each growing vessel contained 26 pea seedlings. *Pisum sativum* L. was grown in Hoagland’s nutrient solution for 4 days in a controlled environment: light of 170 µmol/m^2^ intensity, average temperature 21 ± 3 °C, and 16/8 h day/night photoperiod. On the 5th day, the nutrient solutions were supplemented with nanoparticles. The treatments were as follows, CeO_2_ NPs: 100 mg (Ce)/L; CeO_2_ NPs: 200 mg (Ce)/L; ZnO NPs: 100 mg (Zn)/L; ZnO NPs: 200 mg (Zn)/L; two mixtures of CeO_2_ and ZnO NPs: 100 mg (Ce)/L + 100 mg (Zn)/L and 200 mg (Ce)/L + 200 mg (Zn)/L. Plants grown in Hoagland’s nutrient solution served as the control group. In each variant of the experiment, six growing vessels were used. Fresh liquid media were supplied every 48 h. *Pisum sativum* L. was harvested after 12 days of exposure when plants reached the BBCH 15 phenological stage.

### 2.3. Growth Parameters

**Figure 1:**
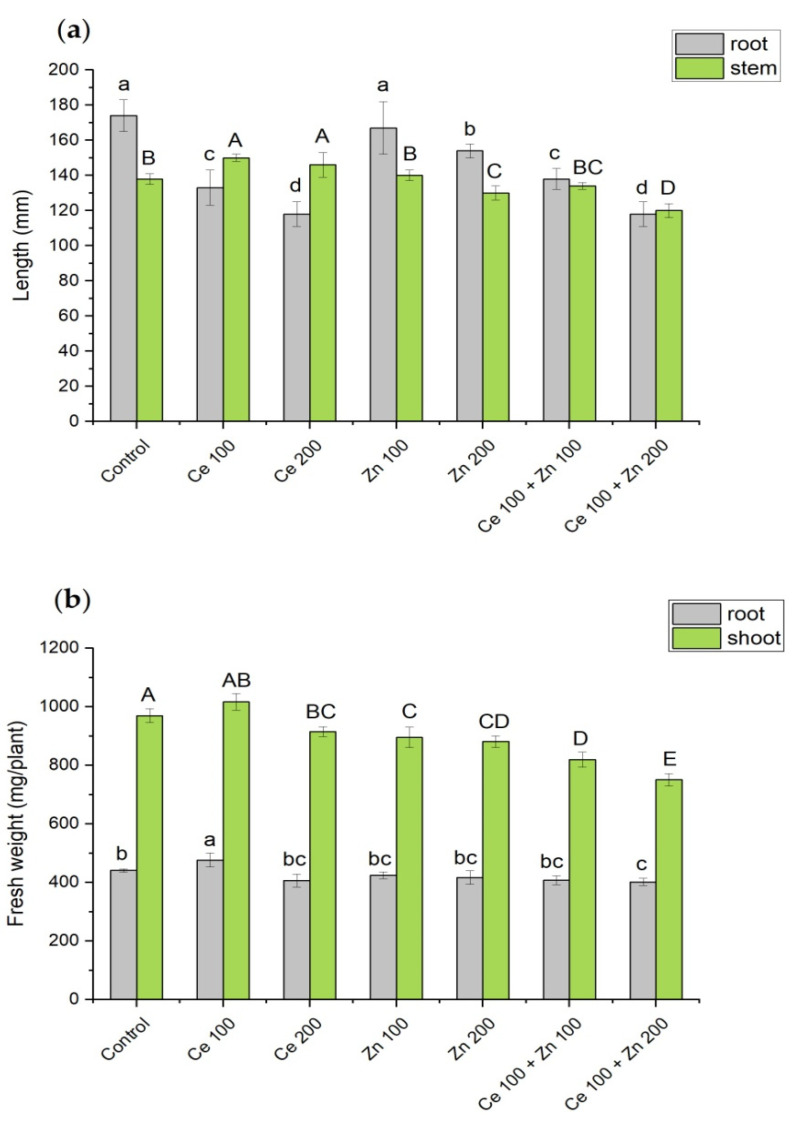
The influence of nanoparticulate CeO_2_ and ZnO on: (**a**) root and stem length and (**b**) fresh weight of root and shoot as determined for a single pea plant. Concentrations of nanoparticles are given in mg/L of elemental cerium or zinc, the cultivation time was 12 days. Roots are represented by grey while above-ground parts are in green. Vertical bars represent standard deviations (*n* = 6). Distinct letters show the statistically significant differences among treatments as calculated with Tukey’s HSD post hoc test. The probability level *p* = 0.95 was applied.

Root and stem lengths were measured at the end of cultivation (Figure 1). Next, the roots were thoroughly rinsed with deionized water and separated from the shoots. The fresh weight of roots and shoots was measured and calculated per single plant (mg/plant). Prior to the chemical analysis, the collected fresh plants material was dried to a constant weight at 55 °C.

### 2.4. Elements Content

The dried samples of roots and shoots were digested in the mixture of concentrated HNO_3_ and HCl (6:1, *v*/*v*) using a Multiwave 3000 Anton Paar microwave reaction system (Anton Paar GmbH, Graz, Austria). The concentrations of Cu, Mn, Zn, Fe, and Mg were determined by a High-Resolution Continuum Source Atomic Absorption spectrometer HR-CS AAS (contrAA300, Analytik Jena, Jena, Germany). Additionally, the digested solutions were analyzed for Ce, Ca, and K concentrations by an Inductively Coupled Plasma–Optical Emission spectrometer ICP-OES (PlasmaQuant PQ 9000, Analytik Jena, Jena, Germany). Certified reference material of plant leaves of Oriental Basma Tobacco Leaves (INCT-OBTL-5) obtained from the Institute of Nuclear Chemistry and Technology, Warsaw [59] was used to check the reliability of the applied analytical procedures. Recoveries ranging from 96% to 108% were obtained. The detailed numerical data are given in Table S2.

### 2.5. Tolerance Index (TI) and Translocation Factor (TF)

Tolerance indices (TI) and translocation factors (TF) were calculated for plants treated with nanoparticulate oxides. TI is defined as the ratio between the root length of treated plants and that of plants in the control group [60,61]. The effectiveness of metal translocation from roots to shoots was assessed using TF, defined as the ratio of average element content in shoots to that in roots [62,63].

### 2.6. Photosynthetic Pigments

Contents of photosynthetic pigments, i.e., chlorophyll a and b and carotenoids in *Pisum sativum* L. leaves were determined following the method developed by Oren et al. [64]. All pigments were extracted from leaves with 80% acetone and kept in the dark at 4 °C. The absorbance of extracts was measured at wavelengths 470, 646, and 663 nm by SpectraMaxi3x (Molecular Devices, San Jose, CA, USA) on samples centrifugated at 4000 rpm for 10 min. Contents of pigments were calculated by the formula proposed by Lichtenthaler and Wellburn [65]. For comparison, the nondestructive measurements of relative chlorophyll content in fully expanded leaves at the fourth node were performed by the Soil Plant Analysis Development SPAD-502Plus chlorophyll meter (Konica–Minolta, Inc., Osaka, Japan).

### 2.7. Photosynthetic Parameters

Photosynthetic parameters, such as: leaf net photosynthesis (A), sub-stomatal CO_2_ concentration (C_i_), transpiration (E), stomatal conductance (gs), photosynthetic water use efficiency (WUE), and photosynthetic CO_2_ response curve (A/C_i_) were determined with a portable gas exchange analyzer (CIRAS-3; PP Systems, Amesbury, MA, USA). Measurements were performed 12 days after the administration of nanoparticles on fully expanded leaves at the fourth node. PARi levels at 1000 µmol/m^2^s were obtained from an LED Light Unit (RGBW) connected to the gas analyzer. The temperature within the chamber was kept at 25 °C, relative humidity at 80%, and reference CO_2_ concentration at 390 µmol/mol. Photosynthetic CO_2_ response curves were collected at a CO_2_ concentration gradient ranging from 0 to 1500 µmol/mol. All measurements were performed on fully expanded leaves at the fourth node. The acclimatization time between measurements was 120 s. The results from each CO_2_ level were recorded three times. Two biochemical parameters: maximum carboxylation rate (V_cmax_) and maximum electron transport rate (*J*_max_) were calculated in Rstudio (v.3.4.2; R Foundation for Statistical Computing, Vienna, Austria) [66] using the “plantecophys” package developed by Duursma [67].

### 2.8. Statistical Analysis

All treatments were replicated six times, numerical results are accompanied by their ± SD (standard deviation). Statistical analysis was performed with the OriginPro 2016 (OriginLab Corporation, Northampton, MA, USA) software. The normality of the sample distributions was proved by the Shapiro–Wilk test [68]. The initial hypothesis on equal variances of investigated populations was validated with the Bartlett test [69]. A one-way ANOVA with Tukey’s post hoc approach was applied to validate differences between means. The probability level *p* = 0.95 was applied.

## 3. Results and Discussion

### 3.1. Growth Parameters

Several plant growth parameters were determined after twelve days of exposure time to illustrate the pea plant behavior under the combined treatments of nanoparticulate CeO_2_ and ZnO. Roots and stem lengths and their fresh weights are shown in Figure 1.

Almost all supplementations decreased roots length of pea as compared to the control treatment. Nevertheless, this effect is less pronounced for plants cultivated with the addition of ZnO (100 and 200 mg/L) than for the CeO_2_ (100 and 200 mg/L) treatments. It is directly correlated with the nanoparticles concentration applied. Notably, supplementation with mixed NPs resulted in roots shortening similar to that observed for relevant sole nanoceria concentrations. The order of tolerance indices (TI) supports above observations: (Zn 100) ≈ (Zn 200) > (Ce 100 + Zn 100) ≈ (Ce 100) > (Ce 200 + Zn 200) ≈ (Ce 200). Numbers in brackets represent supplementations of nanoparticles given in mg/L of elemental cerium or zinc, numerical values are presented in Table S3. Stem elongation was triggered by (Ce 100) and (Ce 200). A reverse effect was observed for (Zn 200) and (Ce 200 + Zn 200) administrations. Notably, the majority of treatments left the root mass unchanged with (Ce 100) and (Ce 200 + Zn 200) being the chief exceptions (Figure 1b). The biomass of the above-ground parts decreased upon all supplementations. The only exception was (Ce 100) with a biomass similar to that of a control sample.

### 3.2. Cerium and Zinc Concentrations

Cerium and zinc concentrations in roots and shoots along with their translocation factors are summarized in Table 1. The highest cerium content was determined for (Ce 200) treatment while the lowest levels were observed for combined (Ce 100 + Zn 100) and (Ce 200 + Zn 200) administrations. Respective translocation factors were remarkably low, which indicates that plants accumulate cerium primarily in roots. Cerium was not detected in plants grown in Hoagland’s nutrient solutions which were not supplemented with this element. Zinc is an essential element necessary for proper plant development [70,71]. The amounts of zinc accumulated by pea cultivated in solutions supplemented with the ZnO NPs alone were substantially larger than those observed for control samples. Combined treatments, namely (Ce 100 + Zn 100) and (Ce 200 + Zn 200) decreased Zn levels in both roots and shoots as compared to respective sole ZnO NPs administrations. However, all relevant concentrations highly exceeded critical toxic zinc level 300 mg/kg as suggested by Broadley et al. [70,71]. Similar to nanoceria administrations, the vast majority of zinc was immobilized in roots.

### 3.3. Photosynthetic Pigments

**Figure 2:**
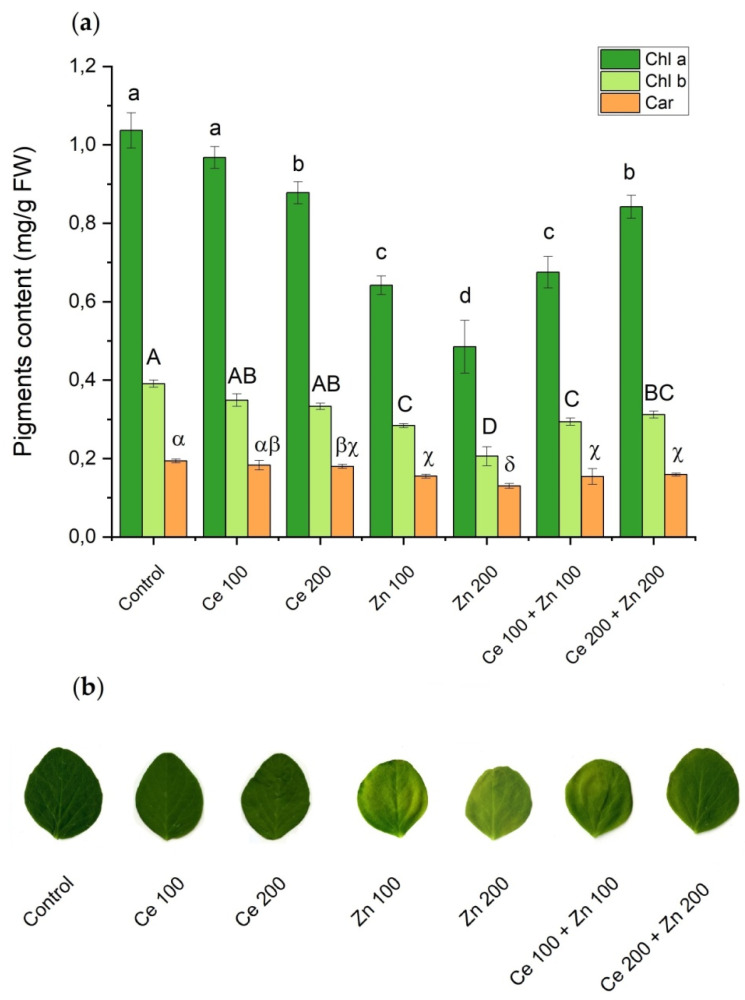
The influence of nanoparticulate CeO_2_ and ZnO on: (**a**) photosynthetic pigments: chlorophyll a (Chl a), chlorophyll b (Chl b), and carotenoids (Car) in *Pisum sativum* L. The pigments were extracted from mature leaves after 12 days of contact with a particular nano-oxide. Vertical bars represent standard deviations (*n* = 6). Treatments with the same letter are not significantly different according to Tukey’s post hoc test (*p* = 0.95) (**b**) appearance of pea leaves from each treatment.

Measurements of photosynthetic pigments provide useful information on the physiological status of plants [72]. Concentrations of chlorophylls and carotenoids which are involved in the absorption and further transfer of light energy are prone to changes induced by inorganic chemical stressors [73]. Contents of chlorophyll a (Chl a), chlorophyll b (Chl b), and carotenoids (Car) in leaves of pea treated with nano-oxides are summarized in Figure 2a.

Significant differences among those parameters were observed. Almost all treatments prompted a decrease in photosynthetic pigments. The highest reduction was observed under (Zn 200) administration. Similar results were obtained by Hu et al. [74] who found that the lowest level of photosynthetic pigments in *Salvinia natans* grown in hydroponic conditions was reached in cultures supplemented with nanometric ZnO at 50 mg/L. Pea plants exposed to (Zn 100) and (Zn 200) showed initial symptoms of leaf chlorosis (Figure 2b). The chlorophyll loss associated with chlorosis is one of the typical symptoms of Zn excess in plants [75,76,77]. In this study, Zn levels in pea shoots under (Zn 100) and (Zn 200) administrations (Table 1) were significantly higher than the critical Zn toxicity level (>300 mg/kg) as quoted in the highly respected Marschner’s Mineral Nutrition of Higher Plants [71].

Moreover, the lowest Chl a, Chl b, and Car concentrations were observed for the sole (Zn 200) treatment. Acute effects of nanoparticulate (Zn 200) were reduced by the (Ce 200) addition. It prompted the mutual, combined interactions which elevated photosynthetic pigments levels and significantly relieved leaf chlorosis symptoms. Following Wang et al. [75] and Broadley et al. [70], Zn may induce chlorosis of leaves by stimulating Mg, Fe, and Mn deficiency. These elements are crucial in the synthesis and stability of chlorophyll. A decline of green photosynthetic pigments in plants under ZnO NPs treatment was also reported by Zoufan et al. [78] who explained it by peroxidation of the chloroplast membrane due to exacerbation of oxidative stress. Oxidative damage triggered by ZnO NPs was also reported by Salehi et al. [79]. In turn, CeO_2_ NPs have the ability to quench ROS, mainly produced in chloroplasts [80,81,82], and presumably mitigate stress induced by nanoparticulate ZnO.

**Figure 3:**
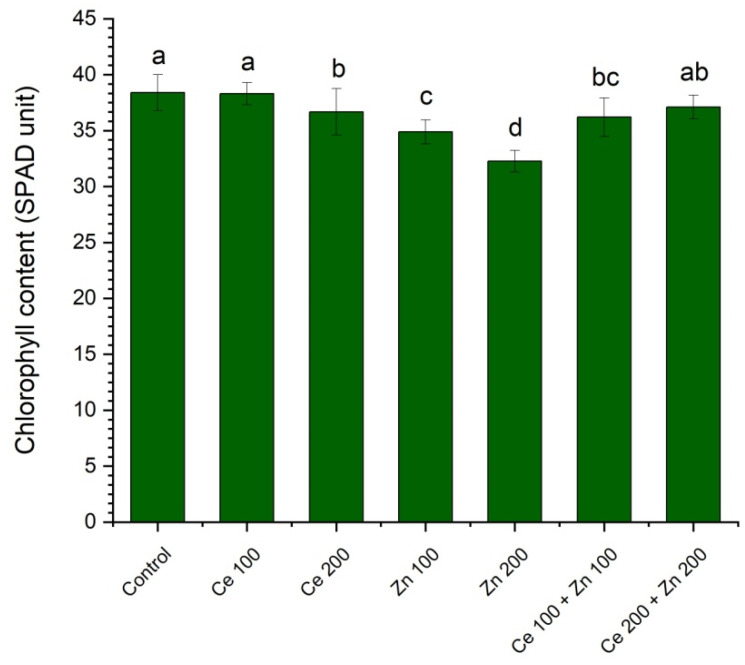
The influence of nanoparticulate CeO_2_ and ZnO on chlorophyll content in *Pisum sativum* L. expressed in SPAD units. All measurements were performed on mature leaves on the 12th day from administration of the nanoparticles. Vertical bars represent standard deviations (*n* = 10). Treatments with the same letter are not significantly different according to Tukey’s post hoc test (*p* = 0.95).

Apart from the conventional wet chemical methods based on extraction of the chlorophyll pigments and their further spectrophotometric determination, the nondestructive measurements using the Soil Plant Analysis Development SPAD-502Plus chlorophyll meter were also performed (Figure 3). The results obtained by all those methods are highly correlated. The reduction in chlorophyll content expressed in SPAD units in *Pisum sativum* L., cultivated in soil supplemented by nanoparticulate ZnO, was also observed by Mukherjee et al. [38], and following Küpper et al. [83] was attributed to the Mg substitution by Zn.

### 3.4. Photosynthesis Parameters

**Figure 4:**
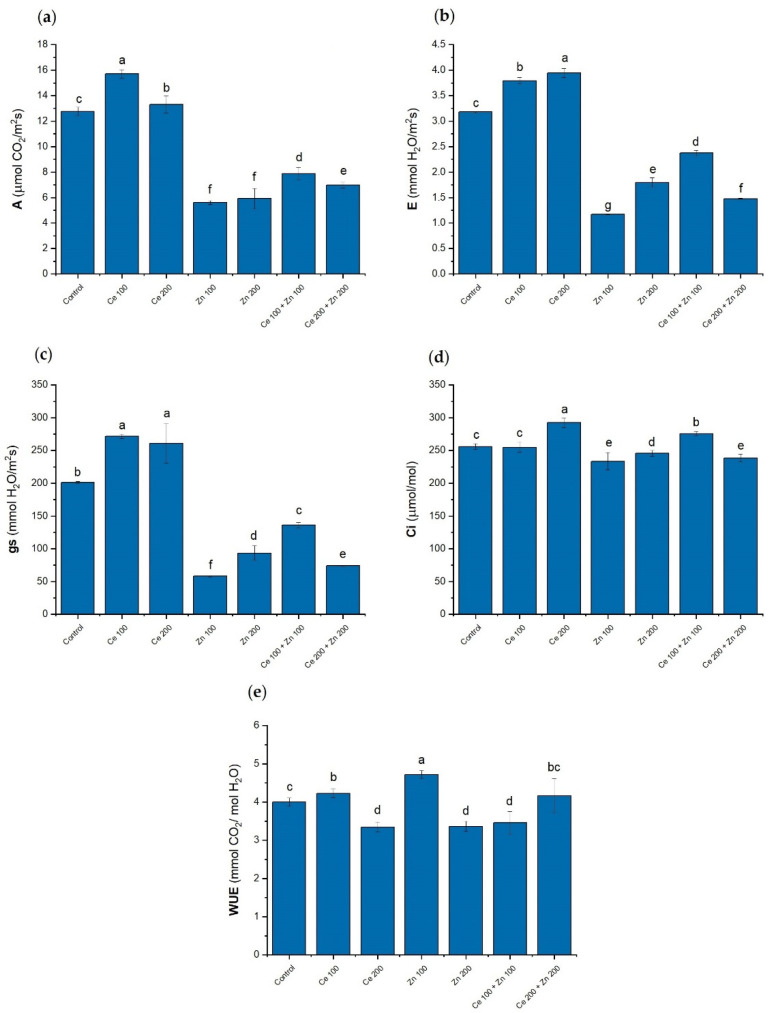
Leaf net photosynthesis A (**a**), transpiration E (**b**), stomatal conductance gs (**c**), sub-stomatal CO_2_ concentration Ci (**d**), and water use efficiency (WUE) (**e**) for *Pisum sativum* L. grown under the sole or combined CeO_2_ and ZnO NPs treatments. Concentrations of nanoparticles are given in mg/L of elemental cerium or zinc, respectively. The cultivation time was 12 days. Vertical bars represent standard deviations (*n* = 6). Distinct letters show the statistically significant differences among treatments as calculated with Tukey’s HSD post hoc test (*p* = 0.95).

Photosynthesis efficiency is usually assessed with the gas exchange analysis of plant leaves [84,85,86]. Leaf net photosynthesis (A), transpiration rate (E), stomatal conductance (gs), sub-stomatal CO_2_ concentration (C_i_), and water use efficiency (WUE) determined under either sole or combined nanoparticles treatments are collected in Figure 4. Net photosynthesis was encouraged by CeO_2_ supplementations and significantly reduced by ZnO additions, Figure 4a. The latter follows reduced chlorophyll content as induced by elevated zinc concentrations [75,87]. This effect is partially released by the nanoceria addition in combined (Ce 100 + Zn 100) and (Ce 200 + Zn 200) treatments. Zinc is an essential metal necessary for proper plant development [70]. However, above certain concentrations, it affects chlorophyll synthesis and photosystem II efficiency [88,89]. Transpiration and stomatal conductance behave in a similar way with the highest values observed for (Ce 100) and (Ce 200) treatments and their substantial decline induced by either (Zn 100) and (Zn 200) supplementations (Figure 4b,c). The sub-stomatal CO_2_ concentrations exhibit a more uniform pattern and are significantly less prone to ZnO supplementations (Figure 4d). The largest water use efficiency was observed for (Ce 100) and (Zn 100) treatments, Figure 4e. High concentrations of (Ce 200) and (Zn 200) nanoparticles prompted a decrease in WUE. Maximum rates of ribulose 1,5-bisphosphate (RuBP) carboxylation as catalyzed by Rubisco (V_cmax_), electron transfer driving the regeneration of RuBP (*J*_max_), and CO_2_ compensation points for pea plants cultivated in Hoagland’s solution supplemented with either sole or combined CeO_2_ and ZnO NPs are in Table 2 and Supplementary Figure S1. The highest V_cmax_ values were observed for the sole nanoceria augmentations [(Ce 100) and (Ce 200)] while the strongest decreases were induced by the sole (Zn 100) and (Zn 200) NPs additions. The electron transfer rates *J*_max_ followed patterns observed for the RuBP regeneration. The lowest carbon dioxide compensation point was determined for the (Ce 100) treatment, the largest was obtained for (Zn 200).

Nanoceria is an effective free radicals scavenger and mimics the activity of several enzymes, namely superoxide dismutase (SOD), catalase (CAT), and oxidase (OXD) [32,90,91,92]. In this respect, it was classified by Korsvik et al. [93] as the first antioxidant nanoenzyme [90]. Stimulation of photosynthesis under abiotic stress mitigated by nanoceria in *Arabidopsis thaliana* was investigated by Wu et al. [32,94]. They pointed out that nanoparticulate CeO_2_ reduced the stress induced by reactive oxygen species, namely ^●^OH, which are not tackled by the usual plant enzymatic scavenging pathways. Those NPs had entered chloroplasts through the nonendocytic pathways, reduced the ROS concentrations, and finally increased the quantum yield of photosystem II, carbon assimilation rates, and chlorophyll content. The latter processes are governed by the reversible redox reactions between the Ce^4+^ and Ce^3+^ species which are followed by the oxygen vacancy generation or elimination [95,96,97]. Moreover, Cao et al. [22,98] identified a strong correlation between photosynthesis parameters and the applied doses of CeO_2_ NPs in soybean. The resulting photosynthesis enhancement was explained by the elevated Rubisco activity (V_cmax_) and promotion of the NADPH regeneration rate which prompted the RuBP synthesis.

Nanometric ZnO affected the CO_2_ assimilation process in a roughly opposite way than CeO_2_ NPs. At low concentrations, zinc is a micronutrient necessary for proper plant development. At elevated levels (above 300 µg/g, plant dry weight), zinc becomes a toxic pollutant responsible for the generation of ROS [99,100]. It alters stomata morphology and formation. The decreased gs and E indicated an increased stomatal closure and restriction of the transpiration rate. Carbon dioxide enters pea leaves by diffusion through stomatal pores. The major photosynthesis limitations result either from diffusion-controlled restrictions in CO_2_ supply to the carboxylation sites or reduction in its consumption through the mitigated Rubisco activity and RuBP regeneration [101]. The decrease in the net photosynthesis A was correlated with the intercellular CO_2_ concentration C_i_ and accompanied by the simultaneous reduction in V_cmax_ and *J*_max_. These results suggest that ZnO NPs induced either stomatal or biochemical photosynthesis limitations. In the mixed ZnO and CeO_2_ treatments, the latter effects are attenuated by the free radical scavenging properties of nanoceria.

### 3.5. Elements Content

**Figure 5:**
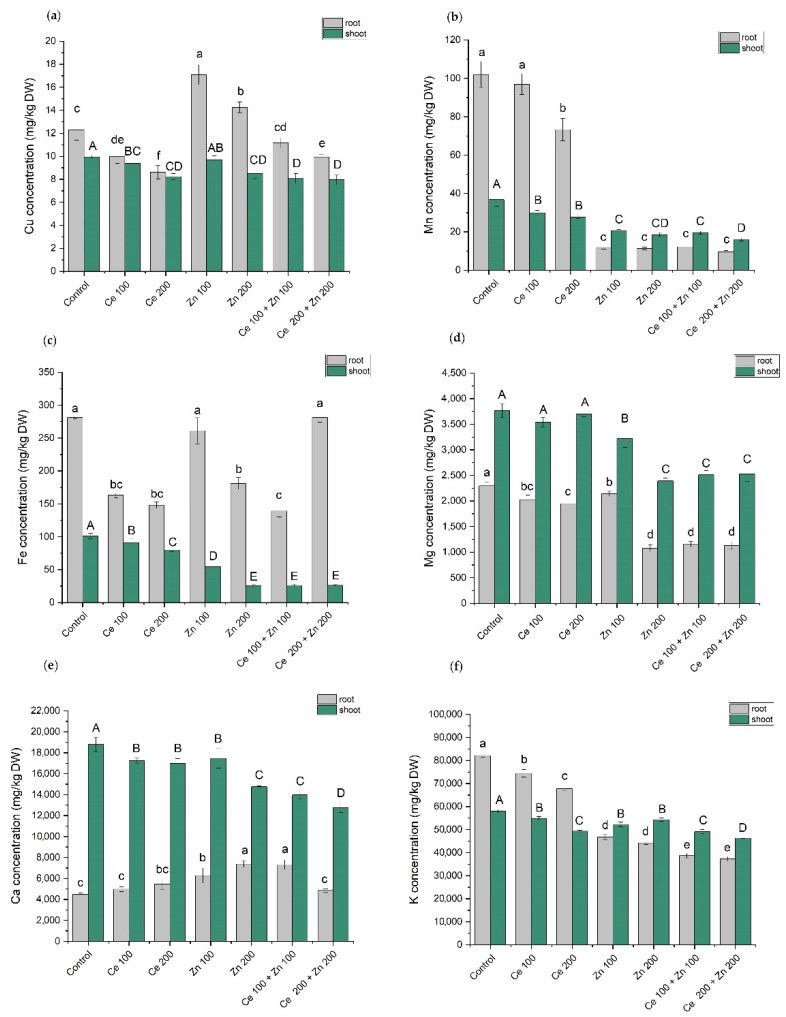
Copper (**a**), manganese (**b**), iron (**c**), magnesium (**d**), calcium (**e**), and potassium (**f**) concentrations in roots and shoots of *Pisum sativum* L. grown under the sole or combined CeO_2_ and ZnO NPs treatment. Concentrations of nanoparticles are given in mg/L of elemental cerium and zinc, respectively. The cultivation time was 12 days. Vertical bars represent standard deviations (*n* = 6). Distinct letters show the statistically significant differences among treatments as calculated with Tukey’s HSD post hoc test (*p* = 0.95).

Copper, manganese, iron, magnesium, calcium, and potassium contents were determined in plants from all treatments. The results are presented in Figure 5 and Table S4. Roots and shoots were treated separately. A one-way ANOVA was initially used to evaluate concentrations of macro- and micronutrients in pea plants. The 0.95 probability level was applied. Both nano-oxides altered the uptake of elements and their further translocation to the green parts of pea (Table S5).

Nanoceria in both tested concentrations [(Ce 100) and (Ce 200)] significantly reduced the uptake of all three investigated heavy metals (Cu, Mn, and Fe) by the roots. On the other hand, nanoparticulate ZnO [(Zn 100) and (Zn 200)] behaved in a less obvious way. Roots uptake was increased for copper and decreased for manganese. The uptake of iron by the roots was not affected by (Zn 100) and was reduced by high zinc supplementation (Zn 200). The combined treatments, either (Ce 100 + Zn 100) or (Ce 200 + Zn 200), prompted more complex root responses. For Cu, the resulting uptakes were between those induced by the administration of either CeO_2_ or ZnO alone. The manganese levels were as low as those observed for the sole (Zn 100) and (Zn 200) supplementations. In the combined (Ce 200 + Zn 200) treatment Fe root level was as high as that in the control sample. However, (Ce 100 + Zn 100) treatment prompted much lower Fe concentrations. In green parts of the pea plants, the levels of Cu, Mn, and Fe were lower than those in roots. The only exception was manganese whose concentrations in shoots were higher for all samples supplemented with nanometric ZnO. Zinc hampers manganese uptake by roots. The latter element is essential for the water-splitting process during the light-dependent phase of photosynthesis. These circumstances facilitate Mn migration from roots to shoots. A quite similar situation was observed for magnesium and calcium. The former is an important chlorophyll cofactor while the latter is a structural component of photosystem II. Both ions are crucial for the overall photosynthesis yield. The additions of (Ce 100) and (Ce 200) NPs alone decreased potassium levels in roots and shoots. Both were inversely proportional to the Ce supplementations. The nanometric ZnO in either sole or combined administrations further restrained potassium contents in pea roots. However, unlike in the case of the nanoceria additions, higher K levels were observed in shoots.

## 4. Conclusions

Nanomaterials alter plant metabolism in a number of ways with photosystems I and II being their important targets. This paper continues our investigations on plant metabolism and uptake of nutrients in a model hydroponic environment subjected to nanoparticles pollution. Steadily increasing usage of nanomaterials in smart agriculture prompts thorough studies on their interactions with plant organisms. Nanoparticles rarely exist in the environment alone and their combined interactions can hardly be neglected.

Regrettably, relevant studies on their impact on plant photosynthesis are scarce. In this study, pea plants were cultivated in Hoagland’s solutions supplemented with either sole or mixed cerium and zinc nano-oxides at 100 mg/L or 200 mg/L Ce or Zn levels. Despite relatively high sole CeO_2_ administration (200 mg/L), no morphological symptoms of phytotoxicity were detected in *Pisum sativum* L. Leaf net photosynthesis, water use efficiency, and fresh biomass production were stimulated at the 100 mg/L Ce concentration and only slightly suppressed at its higher level. Contrarily, ZnO NPs applied alone caused serious impairment of metal homeostasis, decreased the level of photosynthetic pigments, induced leaf chlorosis, and finally hampered photosynthetic efficiency. We proved that ZnO NPs induced stomatal and biochemical limitations of photosynthesis. Such dysfunctions could lead to the overproduction of ROS in chloroplast and induce oxidative stress. In mixed CeO_2_–ZnO NPs treatments, the beneficial effect of nanoceria was observed. In particular, pigments concentrations, leaf net photosynthesis, and maximum electron transport rate (*J*_max_) depressed by ZnO NPs were significantly alleviated when CeO_2_ NPs were present in the growing medium. It is well recognized that nanoceria has the potential to quench ROS. Therefore, we conclude that CeO_2_ NPs moderate ZnO NPs toxicity by protecting the photosynthetic apparatus in *Pisum sativum* leaves from oxidative stress trigged by Zn. Additionally, we observed that both nano-oxides affected the nutrients uptake and transport at all concentrations applied.

Reactive oxygen species are by-products of aerobic metabolic processes in plants [102,103]. They usually increase membrane permeability and initiate stress signals often leading to programmed cell death [104]. At certain concentrations, the presence of NPs in growing media elevates ROS levels and induces oxidative damage in plant species. On the other hand, plant organisms have developed advanced antioxidant systems which involve either enzymatic or non-enzymatic pathways stabilizing ROS levels [105]. Those systems are enhanced under exposure to NPs, perhaps as an adaptive response to alleviate stress. We speculate that either CeO_2_ or ZnO nanoparticles trigger oxidative stress in pea but only cerium dioxide acts as an antioxidant and reduces the stress symptoms while zinc oxide is mainly a prooxidant.

Our future studies will be aimed at the binary activity of nanoceria in agricultural plants. Namely, at high concentrations, it is a plant stressor that triggers ROS production while at certain, low levels nanoceria exhibits a ROS scavenging ability and supports the plant’s defense mechanisms. The latter effect may find applications in agriculture and deserves further investigation.

## Figures and Tables

**Table 1:** Zinc and cerium concentrations in root and shoot of *Pisum sativum* L. grown under the sole or combined CeO_2_ and ZnO NPs treatments accompanied by translocation factors (TF). Concentrations of nanoparticles are given in mg/L of elemental cerium or zinc, respectively. The cultivation time was 12 days. Data are the means ± SD (*n* = 6). Distinct letters show the statistically significant differences among treatments as calculated with Tukey’s HSD post hoc test (*p* = 0.95). nd: concentration below the detection limit (18 µg/L).

Treatment	Zinc Concentration (mg/kg DW)		TF	Cerium Concentrations (mg/kg DW)		TF
	Root	Shoot		Root	Shoot	
Control	97.8 ± 4.5 d	61.4 ± 1.1 d	0.63	nd	nd	nd
Ce 100	74.3 ± 5.5 d	52.4 ± 2.3 d	0.71	14,874 ± 495 b	110 ± 11 c	0.01
Ce 200	68.3 ± 6.2 d	51.5 ± 2.2 d	0.75	16,977 ± 1067 a	206 ± 14 a	0.01
Zn 100	25,921 ± 2307 b	1160 ± 61 bc	0.04	nd	nd	nd
Zn 200	30,522 ± 2140 a	1455 ± 54 a	0.05	nd	nd	nd
Ce 100 + Zn 100	18,192 ± 703 c	1079 ± 113 c	0.06	6058 ± 824 d	120 ± 7 bc	0.02
Ce 200 + Zn 200	28,097 ± 3773 ab	1236 ± 88 b	0.04	10,301 ± 1484 c	142 ± 10 b	0.01

**Table 2:** Maximum ribulose 1,5-bisphosphate carboxylation rates (V_cmax_), maximum electron transport rates (*J*_max_) and CO_2_ compensation points determined for *Pisum sativum* L. plants grown under the sole or combined CeO_2_ and ZnO NPs treatments. Distinct letters show the statistically significant differences among treatments as calculated with Tukey’s HSD post hoc test (*p* = 0.95), *n* = 6.

Treatment	V_cmax_ (µmol/m^2^ s)	*J*_max_ (µmol/m^2^ s)	CO_2_ Compensation Point (µmol/mol)
Control	62.22 ± 1.22 c	80.31 ± 0.93 b	57.40 ± 1.12 b
Ce 100	71.07 ± 1.30 a	87.84 ± 0.69 a	43.00 ± 0.62 c
Ce 200	66.39 ± 0.58 b	85.02 ± 1.10 a	58.84 ± 1.00 b
Zn 100	50.29 ± 1.07 d	73.96 ± 1.27 c	97.51 ± 0.91 a
Zn 200	50.21 ± 0.82 d	67.82 ± 1.00 c	97.95 ± 1.28 a
Ce 100 + Zn 100	60.75 ± 1.41 c	79.09 ± 1.01 b	96.91 ± 1.34 a
Ce 200 + Zn 200	53.81 ± 2.58 d	72.78 ± 1.80 c	57.39 ± 3.27 b

## Data Availability

The data presented in this study are available on request from the corresponding author.

## Supplementary Materials

The following are available online at https://www.mdpi.com/article/10.3390/cells10113105/s1, Table S1: Characterization of CeO_2_ NPs and ZnO NPs, Table S2: Elements content in certified reference materials Oriental Basma Tobacco Leaves—INCT-OBTL-5 (*p* = 0.95, *n* = 8), Table S3: Root length and tolerance index (TI) of *Pisum sativum* L. treated with nanoparticulate CeO_2_ and ZnO, Table S4: ANOVA parameters for elements concentrations in roots and shoots of *Pisum sativum* L. plants across all treatments., Table S5: Translocation factors (TF) of nutrients from root to shoot in *Pisum sativum* L. plants grown under the sole or combined CeO_2_ and ZnO NPs treatments, Figure S1: Comparison of CO_2_ response curves (A/Ci) between control and treated plants.
