# Supplementary material for: The Combined Effect of ZnO and CeO2 Nanoparticles on Pisum sativum L.: A Photosynthesis and Nutrients Uptake Study
Source: Cells. 2021 Nov 10;10(11):3105. doi: 10.3390/cells10113105 (PMC8624121; doi:10.3390/cells10113105)
Supplement: Supplementary file 1 [file cells-10-03105-s001.zip › cells-1408739-supplementary.pdf]

Supplementary Materials

The combined effect of ZnO and CeO_2 nanoparticles on *Pisum sativum* L. A photosynthesis and nutrients uptake study

Elżbieta Skiba [1,*], Monika Pietrzak [1], Sława Glińska [2] and Wojciech M. Wolf [1]

Supplementary Table S1: Characterization of CeO_2 NPs and ZnO NPs

Supplementary Table S2: Elements content in certified reference material Oriental Basma Tobacco Leaves - INCT-OBTL-5 ($\bar{x} \pm ts_{\bar{x}}$, p = 0.95, n = 8).

Supplementary Table S3: Root length and tolerance index (TI) of *Pisum sativum* L. treated with nanoparticulate CeO_2 and ZnO. Concentrations of nanomaterials are given in mg/L of elemental zinc or cerium, the cultivation time was 12 days. Distinct letters show the statistically significant differences among treatments as calculated with Tukey's HSD post hoc test. The probability level p = 0.95 was applied.

Supplementary Figure S1: Comparison of CO_2 response curves (A/Ci) between control and treated plants. Horizontal reference line is set as the CO_2 compensation point (value of C_i when A=0).

Supplementary Table S4: ANOVA parameters for elements concentrations in roots and shoots of *Pisum sativum* L. plants across all treatments.

Supplementary Table S5: Translocation factors (TF) of nutrients from root to shoot in *Pisum sativum* L. plants grown under the sole or combined CeO_2 and ZnO NPs treatments. Concentrations of nanoparticles are given in mg/L of elemental cerium or zinc, respectively. The cultivation time was 12 days.

Table S1. Characterization of CeO_2 NPs and ZnO NPs [52,54]

Property	CeO_2 NPs	ZnO NPs
TEM images	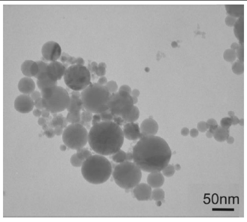	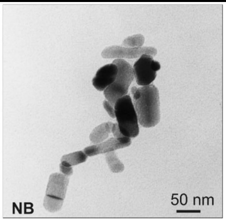
TEM average particle size (nm)	26 ± 14	51 ± 18
Zeta potential (mV)	-41.55 ± 1.13	-3.30 ± 0.29

Table S2. Elements content in certified reference materials Oriental Basma Tobacco Leaves - INCT-OBTL-5 ($\bar{x} \pm ts_{\bar{x}}$, p = 0.95, n = 8).

Element	Certified value	Determined content	Recovery [%]
	[wt %]		
Ca	3.996 ± 0.142	3.976 ± 0.429	99
K	2.271 ± 0.076	2.175 ± 0.139	96
Mg	0.853 ± 0.034	0.832 ± 0.076	97
Fe*	0.149	0.144 ± 0.009	97
	[mg/kg]		
Ce	2.99 ± 0.18	2.86 ± 0.24	96
Cu	10.1 ± 0.4	10.9 ± 0.1	108
Mn	180 ± 6	174 ± 2	97
Zn	52.4 ± 1.8	51.3 ± 1.1	98

* information value

Table S3. Root length and tolerance index (TI) of *Pisum sativum* L. treated with nanoparticulate CeO_2 and ZnO. Concentrations of nanomaterials are given in mg/L of elemental zinc or cerium, the cultivation time was 12 days. Distinct letters show the statistically significant differences among treatments as calculated with Tukey's HSD post hoc test. The probability level p = 0.95 was applied.

Treatment	Root length (mm)	TI
Control	174 ± 9 a	1.00
Ce 100	133 ± 10 c	0.76
Ce 200	118 ± 7 d	0.68
Zn 100	167 ± 15 a	0.93
Zn 200	154 ± 4 b	0.89
Ce 100 + Zn 100	138 ± 6 c	0.79
Ce 200 + Zn 200	118 ± 7 d	0.68

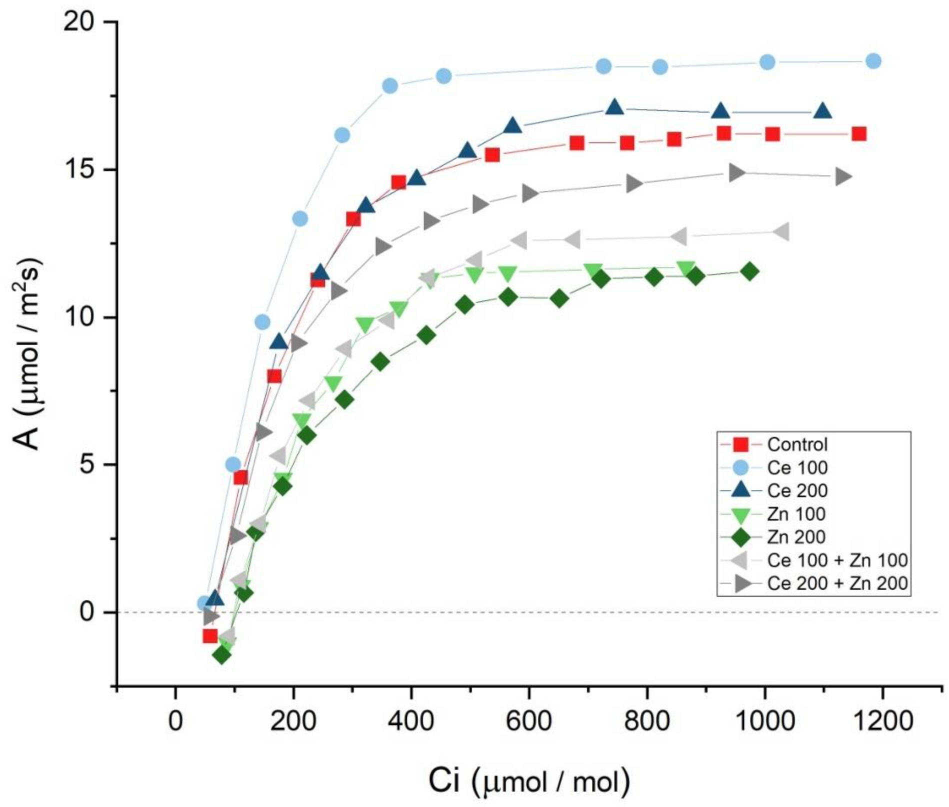

Figure S1. Comparison of CO_2 response curves (A/C_i) between control and treated plants. Horizontal reference line is set as the CO_2 compensation point (value of C_i when A=0).

Table S4. Translocation factors (TF) of nutrients in *Pisum sativum* L. plants grown under the sole or combined CeO_2 and ZnO NPs treatments. Concentrations of nanoparticles are given in mg/L of elemental cerium or zinc, respectively. The cultivation time was 12 days.

Treatment	Translocation factor [-]					
	TF_{Cu}	TF_{Mn}	TF_{Fe}	TF_{Mg}	TF_{Ca}	TF_{K}
Control	0.81	0.36	0.36	1.64	4.18	0.71
Ce 100	0.89	0.31	0.56	1.75	3.47	0.74
Ce 200	0.95	0.38	0.54	1.90	3.10	0.73
Zn 100	0.57	1.79	0.21	1.47	2.79	1.12
Zn 200	0.60	1.63	0.14	2.22	2.00	1.23
Ce 100 + Zn 100	0.72	1.61	0.19	2.18	1.91	1.27
Ce 200 + Zn 200	0.80	1.64	0.09	2.24	2.63	1.24

Table S5. ANOVA parameters for elements concentrations in roots and shoots of *Pisum sativum* L. plants across all treatments.

Element	SS_{total}	$MS_{between}$	MS_{within}	F	p-value	Test F
Root						
Cu	292.0678	46.97811	0.318725	147.3939	7.00E-22	2.399
Mn	44226.91	7321.972	11.34898	645.1659	5.38E-27	2.474
Fe	1794.055	286.9953	2.669745	107.4991	1.48E-17	2.790
Mg	8573500	1407852	4860.972	289.6236	1.59E-22	2.474
Ca	39630315	5975744	164167.4	36.40031	1.27E-10	2.528
K	7.74E+09	1.29E+09	1161359	1107.269	3.89E-24	2.599
Shoot						
Cu	18.60384	2.55977	0.124816	20.50828	1.02E-08	2.474
Mn	1794.055	286.9953	2.669745	107.4991	1.48E-17	2.459
Fe	21684.22	3581.753	12.10654	295.8527	1.80E-15	2.741
Mg	9922856	1593113	14006.88	113.7379	2.15E-17	2.474
Ca	1,3E+08	20621713	246230.9	83.74949	6.72E-15	2.508
K	5.74E+08	90264489	1413548	63.85671	3.47E-13	2.528
